# Bioaccumulation, Biotransformation and Oxidative Stress of 6:2 Fluorotelomer Sulfonamidoalkyl Betaine (6:2 FTAB) in Earthworms (*Eisenia fetida*)

**DOI:** 10.3390/toxics13050337

**Published:** 2025-04-24

**Authors:** Xinlei Zhang, Mengyao Fang, Zhiyuan Bai, Yulu Zong, Shuyan Zhao, Jingjing Zhan

**Affiliations:** Key Laboratory of Industrial Ecology and Environmental Engineering, Ministry of Education, School of Chemical Engineering, Ocean and Life Sciences, Dalian University of Technology, Panjin 124221, China; 1827070467@mail.dlut.edu.cn (X.Z.); pyyoyo20040325@mail.dlut.edu.cn (M.F.); bzy131419@mail.dlut.edu.cn (Z.B.); zyl9686@163.com (Y.Z.); jingjingzhan@dlut.edu.cn (J.Z.)

**Keywords:** 6:2 FTAB, earthworm, metabolism, gut microorganisms, oxidative stress

## Abstract

As a novel perfluorooctane sulfonate (PFOS) alternative, 6:2 fluorotelomer sulfonamide alkylbetaine (6: 2 FTAB) has been detected in the environment and biotas. However, its behaviors and toxicity in earthworms remain unclear. Here, earthworms (*Eisenia fetida*) were exposed to 6:2 FTAB to investigate its bioaccumulation, biotransformation and toxicity. Results indicated that 6:2 FTAB could be biodegraded in soil into perfluorohexanoic acid (PFHxA), perfluoropentanoic acid (PFPeA), perfluorobutanoic acid (PFBA) and perfluoropropionic acid (PFPrA). The uptake rate constant (*k*_u_) and the bioaccumulation factor (BAF) of 6:2 FTAB in earthworms were 0.0504 g_oc_ g_ww_^−1^ d and 1.65 g_oc_ g_ww_^−1^, respectively. 6:2 FTAB was biotransformed to form PFHxA, PFPeA, PFBA and PFPrA in earthworms after in vivo and in vitro exposure. The aerobic bacteria isolated from worm gut could degrade 6:2 FTAB to form PFPeA and PFHxA, while the anaerobic bacteria did not contribute to 6:2 FTAB biodegradation in worms. Peroxidase (POD) and superoxide dismutase (SOD) activities were significantly increased, while no significant changes were observed for catalase (CAT) activities, demonstrating activation of the primary antioxidant defense system against oxidative stress in earthworms after exposure to 6:2 FTAB. The significant increase of glutathione-S-transferase (GST) activities suggested indirect evidence on the conjugation of 6:2 FTAB or its metabolites in phase II of detoxication. This study provides important information on the fate of 6:2 FTAB in earthworms.

## 1. Introduction

Per- and polyfluoroalkyl substances (PFASs) are widely used in industrial production, commercial applications, and consumer products due to their strong chemical stability, excellent thermal stability, surface activity, and hydrophobic and oleophobic properties [1,2]. However, PFASs have been recognized as environmental pollutants due to their environmental persistence, bioaccumulation, and biological toxicity [3]. Perfluorooctanoic acid (PFOA) is a representative compound of perfluoroalkyl carboxylic acids (PFCAs) which was discontinued for production in 2015 [4,5,6]. Perfluorooctane sulfonate (PFOS), a typical representative of perfluoroalkyl sulfonic acids (PFSAs), has been listed in Stockholm Convention Persistent Organic Pollutants (POPs) in 2009 and gradually phased out. The phase out of long-chained PFCAs and PFSAs resulted in the necessity for alternatives [2].

The 6:2 fluorotelomer sulfonamide alkylbetaine (6:2 FTAB, C_15_H_19_F_13_N_2_O_4_S) is a novel substitute for PFOS, which is used as a surfactant in Aqueous Film-Forming Foams (AFFFs). Recent studies have shown the detection of 6:2 FTAB in various environmental matrices associated with the production or use of AFFF [7]. 6:2 FTAB has been detected at a concentration of 968 ng L^−1^ near discharge points of wastewater treatment plants (WWTPs) in northern France [8]. Water samples collected near an Air Force base revealed that 6:2 FTAB levels ranging from 76 to 1774 ng/L in civilian airport areas, while concentrations near fuel storage facilities were significantly higher, measuring between 4267 and 5350 ng L^−1^ [9]. 6:2 FTAB has been identified in surface soils at firefighting training grounds, reaching concentration as high as 631,338 ng g^−1^. It has been reported that 6:2 FTAB had lower bioaccumulation ability in zebrafish than that of fluorotelomer sulfonamide alkylamine (6:2 FTAA), and induced apoptosis, oxidative stress and immunotoxicity in zebrafish embryos [10]. Research also indicated that 6:2 FTAB was metabolized in both blue mussels and Atlantic halibut, with exposed juvenile halibut showing immune suppression at the transcriptional level, along with altered leukocyte differentiation profiles [10]. Our previous study found that 6:2 FTAB could be taken up by plants and biotransformed to terminal PFCAs, and caused the damage of antioxidant defense systems [11]. However, the behaviors and toxicity of 6:2 FTAB to terrestrial invertebrates remain unclear.

Earthworms, which are widely distributed and constitute a significant proportion of soil biomass, play a crucial role in soil ecosystems and serve as valuable indicators for assessing the bioavailability and toxicity of xenobiotics in terrestrial environments [12]. Metabolic biotransformation of xenobiotics in earthworms may involve both enzymatic metabolism in tissues and microbial degradation in the gut. Cytochrome P450 enzymes (CYP450) are involved in phase I biological metabolism and detoxification of xenobiotics, while glutathione S-transferase (GST) are phase II metabolic enzymes that catalyze conjugation reactions to make the conjugates more readily excretable, achieving detoxification [13]. The earthworm gut contains a unique microenvironment with a large number of anaerobic and aerobic bacteria, which contribute to the degradation of some organic pollutants within the earthworms, such as hexachlorocyclohexane (HCH) and phosmet [14]. According to our previous study, 6:2 fluorotelomer sulfonic acid (6:2 FTSA), a common PFOS alternative, could be bioaccumulated in earthworms and then biotransformed by enzymes (CYP450 and GST) to stable products, such as PFHxA, PFPeA, PFBA, PFPrA and trifluoroacetic acid (TFA) [15]. Both enzymes (CYP450 and GST) and gut aerobic microorganisms contributed to 6:2 fluorotelomeric carboxylic acid (6:2 FTCA) biotransformation in earthworms [15], while the biotransformation of perfluorooctane sulfonamide (PFOSA, a PFOS precursor) in earthworms was mediated mainly by enzymes rather than by gut microbes [16]. Additionally, the bioturbation behavior of earthworms can optimize the redox microenvironment of soil, creating conditions for anaerobic/aerobic microbial degradation of PFASs [17]. Due to the widespread occurrence and high environmental concentrations of 6:2 FTAB in soil, it can be taken up by earthworms, and then biotransformed to other PFASs, which play important roles in 6:2 FTAB fate in soil-earthworms systems. Thus, 6:2 FTAB poses a serious environmental threat to earthworms. However, the bioaccumulation, biotransformation and the consequent ecotoxicological responses of 6:2 FTAB in earthworms are still unknown.

Hence, in the present study, earthworms (*Eisenia fetida*) were exposed to the spiked soil to investigate the bioaccumulation, metabolic transformation and toxicological effects of 6:2 FTAB in earthworms after in vivo and in vitro experiments. In addition, the ability of earthworm gut bacteria, including aerobic and anaerobic bacteria to transform 6:2 FTAB, was explored by inoculating the bacterial colonies in a simulated intestinal environment. The activities of antioxidant enzymes, including CAT, SOD, POD, and GST, were measured to assess the responses of anti-oxidative enzymes under 6:2 FTAB stress. This research will provide a theoretical basis for accurately assessing the ecological risks of 6:2 FTAB in the environment.

## 2. Materials and Methods

### 2.1. Reagents and Materials

6:2 FTAB (98%), perfluoropentanoic acid (PFPeA, 97%) was purchased from J&K Chemical Ltd. Perfluorohexanoic acid (PFHxA, 98%) was obtained from Matrix Scientific. Perfluoropropanoic acid (PFPrA, 97%) and perfluorobutanoic acid (PFBA, 98%) were from Shanghai Macklin Biochemical Technology Co., Ltd. Nicotinamide adenine dinucleotide (NADH) and β-nicotinamide adenine dinucleotide phosphate (NADP^+^) were purchased from Biosharp (Hefei, China). Glucose-6-phosphate dehydrogenase (G6PDH) and glucose-6-phosphate sodium (GLC-6-PO4) were obtained from Sigma-Aldrich (China). HPLC-grade methanol (99.9%) and methyl tert-butyl ether (MTBE) were purchased from Dalian Bonno Biochemical Reagents Co., Ltd. (Dalian, China). Milli-Q water (18.2 MΩ·cm, TANKPE060) was used throughout the experiments.

### 2.2. In Vivo Exposure Experiment

The test soil was collected from the topsoil (0–10 cm) of farmland in Panjin, Liaoning Province. After naturally air-drying for 14 d, the soil was sieved through a 2-mm mesh. A prepared stock solution of 6:2 FTAB in methanol was spiked in the soil and mixed thoroughly, then equilibrated in a fume hood at room temperature for 4 d [18]. The initial exposure concentration of 6:2 FTAB in the experimental soil was measured to be 62.0 ng g^−1^ dry weight (dw), which was based on the median concentration of 6: 2 FTAB detected in soil. Earthworms (*Eisenia fetida*) were purchased from a worm farm (Shenyang, China) and acclimatized for 2 weeks at room temperature (22–27 °C). Ten mature earthworms with reproductive clitellum were selected after acclimatization and depuration for 24 h before being placed into a beaker containing 100 g of contaminated soil (with a moisture content of 30%). A blank control group was set up with earthworms exposed to uncontaminated soil. 6:2 FTAB-spiked soils without earthworms were set up as the earthworm-free controls to investigate the biodegradation of 6:2 FTAB in soil. All the beakers of the treatments (three replicates, *n* = 3) were capped with aluminum foil and cultured in the dark at 22 ± 2 °C for 1, 2, 4, 6, 8, 12, 16 and 20 d. Earthworms were sampled from each beaker on days of 1, 2, 4, 6, 8, 12, 16 and 20 for the uptake phase, and 22, 24, 28, 32, 36, 40 for the elimination phase. After sampling, the earthworms were washed, purged on clean filter paper for 24 h, weighed immediately and stored at −20 °C before analysis.

### 2.3. In Vitro Exposure Experiment

The in vitro metabolism assays were performed by incubating the individual earthworm homogenates on the basis of our previous study [19]. Earthworms which were exposed in non-spiked soil and purged on wet filter paper for 24 h were placed in a glycerol solution (20%) at 0 °C for 15 min to induce euthanasia. The earthworms were homogenized with cold buffer (4 °C, 0.1 M, pH 7.4, containing 1 mM EDTA and 0.15 mM KCl) at a ratio of 4:1 (earthworm:buffer) using a mechanical homogenizer. The incubations were conducted in some polypropylene (PP) tubes (50 mL) wrapped with aluminum foil. The reaction of 6:2 FTAB (50 μL 6:2 FTAB solution in methanol) with homogenates (0.5 g) contained 3.95 mL of 0.05 M phosphate buffer, 500 μL of premixed NADPH regenerating solution (containing 1.6 mM NADP+, 3.3 mM Glc-6-PO4, 0.4 U m L^−1^ G6PDH, and 3.3 mM MgCl_2_). The initial spiked concentration of 6:2 FTAB was set at 20.8 ng g^−1^ wet weight. Reaction mixtures were vortexed and incubated with shaking in a water bath at 25 °C in the dark. Sampling time was chosen at the 0, 2, 4, 8, 12, 24 and 32 h time points during incubations. Three control groups were set up, including control I (homogenate without 6:2 FTAB), control II (6:2 FTAB + homogenates inactivated by boiling at 100 °C for 5 min, excluding the interference of abiotic or microbial) and control III (6:2 FTAB, excluding the photodegradation), with samples collected after 2 h and 32 h of incubation. After incubation, three PP tubes were sacrificed and 2.5 mL of methanol was added to terminate the reaction. After vortexing (1 min), the solution was frozen at −20 °C until extraction and analysis.

### 2.4. Enzyme Assays

Toxicokinetics of 6:2 FTAB in earthworms were conducted during the 20 d uptake phase experiments. The sample of one earthworm (approximately 0.3 g) was homogenized in a manual homogenizer on an ice bath with 2.7 mL phosphate-buffered saline (0.05 mol L^−1^, pH 7.5) [19]. The earthworm homogenate was then centrifuged at 4 °C and 12,000 rpm for 10 min, and the supernatant was collected for the further analysis of CAT, POD, SOD and GST enzyme activities. All the enzymatic activities were determined using a Multi-Mode Microplate Reader (Molecular Device, San Jose, CA, USA) at 450 nm, as described by the instructions of the earthworm ELISA Kits (Dongge Biotechnology Co. Ltd., Beijing, China). Detailed information was shown in the supporting information (SI). GST activity was expressed as nmol min^−1^ mg^−1^ protein, and other enzyme activities (SOD, POD and CAT) were expressed as U mg^−1^ protein.

### 2.5. Biodegradation of 6:2 FTAB by Earthworm Gut Microbes

The assays were performed on the basis of previous studies with slight modifications [20]. Earthworms were acclimated for 20 d in soil spiked with 6:2 FTAB. Afterward, the earthworms were anesthetized, eviscerated and cleaned with 75% alcohol. The gut segment below the stomach was then removed, and a 0.1 g (fresh weight) portion of the gut was homogenized in 1 mL of sterilized pre-cooled PBS (0.1 mol L^−1^, pH 7.0) until no visible particles remained.

An aerobic degradation experiment was conducted under sterile conditions. A total of 1 mL of the earthworm gut homogenate was added to 100 mL of LB liquid culture medium for mixed bacterial enrichment culture (water bath shaker, 100 rpm). Bacterial populations in the logarithmic growth phase were taken from the LB liquid medium for further acclimation, and the second-generation bacterial population was cultured to the logarithmic phase. A total of 50 mL suspension of the second-generation logarithmic phase culture was centrifuged (300 rpm, 10 min) to remove the liquid LB. Then, 0.2 mL of the gut microbia suspension was added to a PP tube containing 6:2 FTAB contaminated MSM medium, mixed thoroughly and incubated under aerobic conditions on a water bath shaker (25 °C, 100 rpm).

An anaerobic degradation experiment was conducted inside an anaerobic glove box. A total of 1 mL of earthworm gut homogenate was added to deoxygenated LB liquid medium for anaerobic incubation. The gas phase of all the incubations was aerated with 100% N_2_ to provide an anaerobic environment. After being removed from the anaerobic glove box, the incubations were placed in a water bath shaker (25 °C, 100 rpm) to culture the second-generation logarithmic phase. After centrifugation, 0.2 mL of the suspension was added to deoxygenated MSM medium (6:2 FTAB) and carried out in an anaerobic glove box for the degradation experiment.

The entire experiment was conducted in the absence of light, with different conditions set: the group with 6:2 FTAB as the sole carbon source (6:2 FTAB + gut microbes, F + M); the group excluding abiotic interference (6:2 FTAB + boiled gut, C); the group with 6:2 FTAB and added carbon source (6:2 FTAB + 0.5% glucose + gut microbes, F + M + G); the blank control group with 6:2 FTAB (6:2 FTAB, F). After incubation for 48 h, the same volume of methanol was added to the PP tubes to terminate incubation, and stored at −20 °C for PFASs extraction and analysis.

### 2.6. Chemical Extraction and Instrumental Analysis

The extraction and purification of soil, earthworm and intestinal microbial samples followed the steps in our previous studies [16], which had been shown in SI. A quantitative analysis of PFASs content in the samples was performed using a Waters UPLC system coupled to a Waters XEVO-TQS tandem mass spectrometry (UPLC-MS/MS) in the negative electrospray ionization (ESI) and multiple reaction monitoring (MRM) mode. The separation of PFASs was achieved with a Waters UPLC C18 chromatographic column (1.7 μm, 2.1 mm × 50 mm) at 38 °C. The mobile phase consisted of an ammonium formate aqueous solution (A, 2 mmol L^−1^) and methanol (B), with a flow rate of 0.45 mL min^−1^ and an injection volume of 10 μL. The gradient elution conditions were as follows: 0–0.5 min, 25% B; 0.5–5.0 min, 25–85% B; 5.0–5.1 min, 85–100% B; 5.1–8.0 min, 100% B; 8.0–10.0 min, 100–25% B. The mass spectrometry conditions were as follows: capillary voltage −2.2 kV, nebulizer gas flow rate 7.00 bar, desolvation gas flow rate 800 L h^−1^, cone gas flow rate 150 L h^−1^, ion source temperature 150 °C and desolvation temperature 400 °C. The quantitative parameters for all PFASs are listed in Table S1.

### 2.7. Quality Assurance and Quality Control

The recoveries were determined using a blank matrix with spiking, and the degradation experiments were conducted using a blank sample prepared with the same method. Additionally, the external standard method with added matrix was used for quantification. The method detection limit (MDL) was based on a signal-to-noise ratio of 3:1. The recoveries of PFASs in various matrices ranged from 81.5% to 107%. The MDLs for PFASs in soil, earthworm, homogenate and gut microbe incubation were 0.0005 to 0.0075 pmol g^−1^ dw, 0.0001 to 0.0020 pmol g^−1^ ww and 0.0012 to 0.0029 pmol mL^−1^, respectively. The recoveries and MDLs of the analyzed PFASs are shown in Table S1.

### 2.8. Data Analysis

The elimination rate constant (*k*_e_) for 6:2 FTAB and its products in earthworms were calculated using a first-kinetic decay through linear fitting (Origin 8.0, Northampton, MA, USA).

(1) $$C_{e}=C_{0}e^{{-k}_{e}t}$$

where *C_e_* and *C*_0_ are the concentrations of 6:2 FTAB and PFCAs detected in earthworms (nmol·g⁻^1^ ww) at time *t* (d) and at the beginning of the depuration, respectively, while *k_e_* is the elimination rate constant of 6:2 FTAB and its metabolites in earthworms (d^−1^).

The depuration half-life (*t*_1/2_) of PFASs was calculated as:

(2) $$t_{1/2}=\frac{\ln2}{k_{e}}$$

One first-order bioaccumulation model was used to estimate the uptake rate constant (*k_u_*, 1/d) for 6:2 FTAB in earthworms. The rate constants were calculated by nonlinear curve fitting (Origin 8.0, USA).

(3) $$C_{e}=\frac{k_{u}C_{S}}{k_{e}}(1-e^{{-k}_{e}t})$$

where *C_s_* is the initial concentration of 6:2 FTAB spiked in soil (nmol g^−1^ dw).

The kinetic bioaccumulation factor (BAF) was calculated as:

(4) $$\mathrm{BAF}=\frac{k_{u}}{k_{e}}$$

### 2.9. Statistical Analysis

The statistical differences between treatments and controls were assessed by analysis of variance ANOVA and the paired-samples *t*-test. Tukey’s Test was used to determine the significant differences in PFASs concentrations in gut microbe incubations. Statistical differences were defined as *p* < 0.05 (IBM SPSS Software, 22.0).

## 3. Results and Discussion

### 3.1. Degradation of 6:2 FTAB in Soil

**Figure 1:**
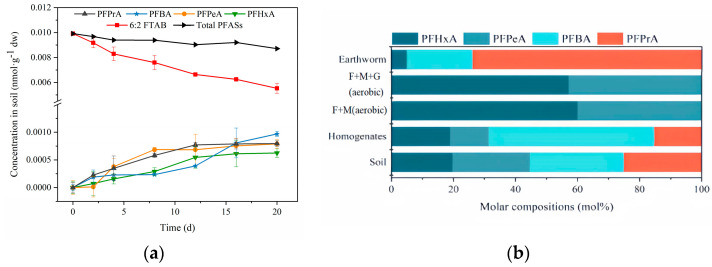
(**a**) Time-dependent concentrations of PFASs in incubation soil; (**b**) PFCA metabolite molar distribution profiles in different experimental groups.

No 6:2 FTAB and PFCAs were detected in the background exposure soil. The exposure concentration was based on the median value of 6:2 FTAB detection concentration in the actual soil. The time-dependent concentrations of 6:2 FTAB and PFCAs products in incubation soil are shown in Figure 1a. Compared to the initial concentration of 6:2 FTAB in the spiked soil, the concentration decreased significantly to 87.1% by day 20. Concurrently, the concentrations of PFPrA, PFBA, PFPeA and PFHxA in the samples increased markedly over time. 6:2 FTAB concentrations in soil could be fitted well by a first-order kinetic model (r^2^ = 0.976, *p* < 0.01). The biodegradation rate constant of 6:2 FTAB in soil (k_s_) was calculated to be 0.0315 d⁻^1^, with a degradation half-life of 22.0 d. Our previous study showed that the half-life of 6:2 FTSA in soil was 11.7 d [15], suggesting that 6:2 FTAB biodegraded more slowly than 6:2 FTSA in soil. It is also reported that the estimated biotransformation half-life of 6:2 FTAB in the petroleum hydrocarbon-contaminated soil was 31 d, assuming first-order kinetics [21]. These results were in contrast to those of Fang et al. [22], who reported that 6:2 FTAB was persistent in aerobic sludge due to its terminal betaine groups in fluorotelomer betaines which were considered to be hardly biodegraded.

As shown in Figure 1b, at the end of the experiment, PFBA, PFPrA, PFPeA and PFHxA were detected in the soil, accounting for 30.5 mol%, 25.0 mol%, 24.8 mol% and 19.7 mol%, respectively, with PFBA being the predominant terminal degradation product. Terminal PFCA products of 6:2 FTAB, including PFBA, PFPeA and PFHxA were observed in sewage treatment plant sludge inoculum [23] and petroleum hydrocarbon-contaminated soil [24] under aerobic conditions. However, no terminal PFCA products were observed in aerobic sludge due to the relative recalcitrance of 6:2 FTAB in aerobic sludge [23].

### 3.2. Uptake and Elimination of 6:2 FTAB in Earthworms

**Figure 2:**
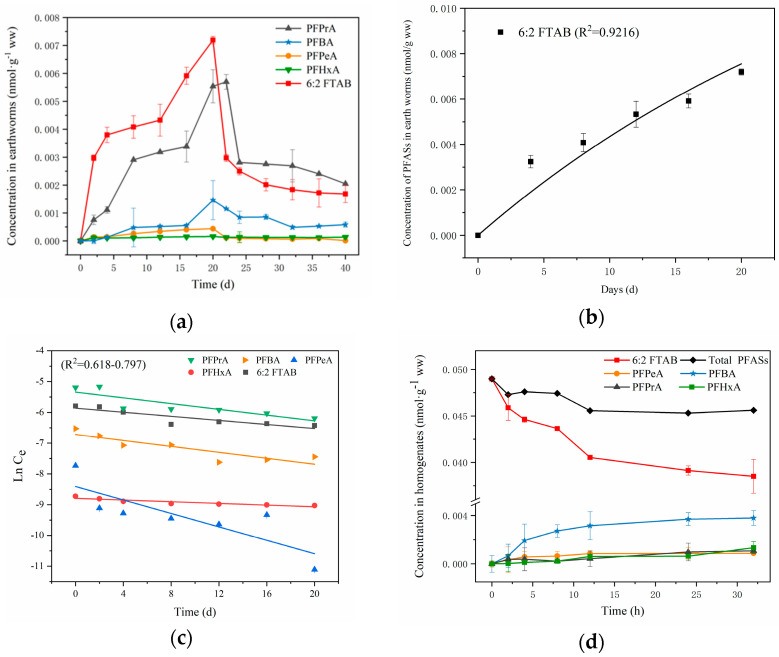
(**a**) The concentrations of 6:2 FTAB and PFCA metabolites in earthworms during uptake and depuration phase; (**b**) uptake kinetic curves fitted by nonlinear regression (*r*^2^ = 0.9216, *p* < 0.01); (**c**) depuration kinetics of PFASs after exposure to 6:2 FTAB in spiked soil for 20 d and then eliminated in clean soil for 20 d (*r*^2^ = 0.678–0.797, *p* < 0.05); (**d**) variation of the concentrations of 6:2 FTAB and its metabolites over time in earthworm homogenates. Values indicate mean ± SD (*n* = 3).

During the uptake and elimination experiment periods, all the test earthworms were in good health. No PFASs were detected in earthworms in the blank control group, suggesting no background contamination was observed during the experimental process. The bioaccumulation of compounds in earthworms is a comprehensive process, including absorption, elimination and biotransformation. The significant accumulation of 6:2 FTAB in earthworms, accompanied by in vivo biotransformation and removal of 6:2 FTAB, were observed in earthworms (Figure 2a). The concentrations of 6:2 FTAB in earthworms increased rapidly on the second day of exposure and reached a peak at the end of the uptake stage (20 d), which led to increases in PFCA products (PFPrA, PFBA, PFPeA and PFHxA), with PFPrA as the main metabolite (Figure 2a).

The first-order bioaccumulation kinetic model fitted the 6:2 FTAB variation in the earthworms well (r^2^ = 0.9216, *p* < 0.01) (Figure 2b). The uptake rate constant (k_u_) and bioaccumulation factor (BAF) of 6:2 FTAB in earthworms were 0.0504 g_oc_ g_ww_*^−^*^1^ d and 1.65 g_oc_ g_ww_*^−^*^1^, respectively, indicating the high bioaccumulative ability of 6:2 FTAB in earthworms. Otherwise, the BAF value of 6:2 FTAB in earthworms was much higher than its legacy PFOS [25], whose BSAF values ranged from 0.097 to 0.145 g_oc_ g_ww_*^−^*^1^. In addition, four PFCAs with different carbon chain lengths, were detected in earthworms. The elimination kinetics of these compounds were indicated in Figure 2c, which could be fitted well by the first order elimination kinetics model (r^2^ = 0.678–0.797, *p* < 0.05). The k_e_ value of 6:2 FTAB, PFPrA, PFBA, PFPeA and PFHxA were 0.030, 0.064, 0.072, 0.093 d and 0.022 d*^−^*^1^; the t_1/2_ values were 22.7, 10.8, 9.7, 7.5 and 31.6 d, respectively. The results showed that the elimination rate in earthworms was PFPeA> PFBA> PFPrA> 6:2 FTAB> PFHxA, and the half-life follows the order: PFHxA> 6:2 FTAB> PFPrA> PFBA> PFPeA. The half life of 6:2 FTAB (22.7) was much higher than its legacy PFOS (18 d) [25]. All the results indicated that 6:2 FTAB had stronger bioaccumulative ability than PFOS in earthworms.

### 3.3. In Vitro Biotransformation of 6:2 FTAB in Earthworm Homogenate

To further verify the biotransformation of 6:2 FTAB in earthworms, an in vitro experiment was carried out which exposed earthworm homogenates to 6:2 FTAB. As shown in Figure S1, no PFASs were detected in control I, no PFCA metabolites except for parent 6:2 FTAB were observed in controls II and III, and no significant differences were observed between the 2 h and 32 h samples. These results suggest that the effects of photodegradation and microbial degradation of 6:2 FTAB could be negligible in the in vitro test period. The time-dependent decrease of parent 6:2 FTAB and the increase of the PFCA metabolites (PFPeA, PFBA, PFPrA and PFHxA) during the incubation are displayed in Figure 2d, suggesting the biotransformation of 6:2 FTAB in earthworm homogenates. At the end of the incubation experiment, the total amount of PFASs in the earthworm homogenates was 87.8% of the amount of 6:2 PFAB added initially, which might be due to the volatilization of some substances, the adsorption on the container, and undetected intermediates (such as 6:2 FTUCA, 6:2 FTOH, 6:2 FTAA, 5:2 sFTOH, etc.) during the experiment. The degradation rate of 6:2 FTAB in earthworm homogenates was 44.2% after 32 h in vitro culture. The degradation kinetics of 6:2 FTAB in in vitro was consistent with the first-order decay kinetic model, and the biotransformation rate constant in earthworm homogenate was 0.0092 h*^−^*^1^ (r^2^ = 0.791, *p* < 0.01). Comparing with our previous study concerning 6:2 FTSA (0.012 h*^−^*^1^) [15], the biodegradation rate of 6:2 FTAB was much lower than 6:2 FTSA, which could be due to the large molecular size of 6:2 FTAB.

**Figure 3:**
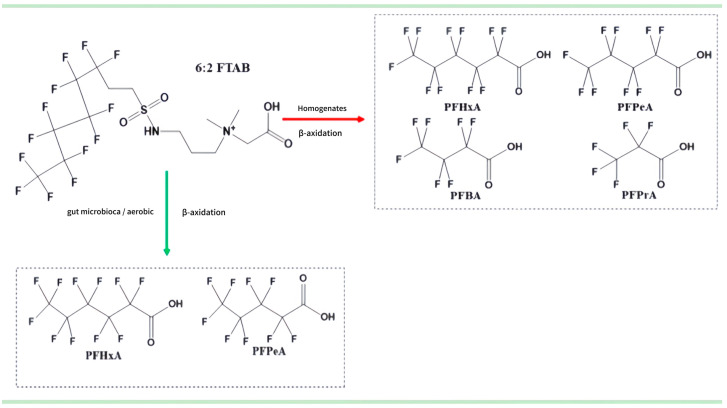
Proposed biotransformation pathways of 6:2 FTAB to terminal PFCA metabolites in earthworm homogenates and gut microorganisms.

According to Figure 1b, four terminal PFCA metabolites were detected in earthworm homogenate with the molar distribution profiles as follows: PFBA (53.5 mol%) > PFHxA (18.9 mol%) > PFPrA (15.2 mol%) > PFPeA (12.3 mol%), suggesting PFAB was the main terminal metabolite. This trend was different from that of 6:2 FTSA biodegradation in earthworms, which yielded TFA (72.0 mol%) as the dominant metabolite [15]. These results suggested that the cleavage of N-S, C-S and C-C bonds of 6:2 FTAB, and β-oxidation and elimination of HF to short chain PFCA products were observed in earthworms. This phenomenon was different from the biotransformation of 6:2 FTAB in plants, which experienced α-oxidation and β-oxidation to form six terminal PFCAs (PFHpA, PFHxA, PFPeA, PFBA, PFPrA) [11]. It was reported that 6:2 FTAB could decarboxylate in mussel and turbot, and then demethylate/deacetylate quaternary amine directly to form 6:2 FTSAm [20]. Based on the above analysis, it could be inferred that the metabolic transformation pathway of 6:2 FTAB in earthworm homogenate was as follows (Figure 3): 6:2 FTAB was transformed to 6:2 FTSAm by removing the betaine group, which was replaced by a carboxyl group, followed by deacidification and N-dealkylation which led to the hydrolysis of the sulfonamide functional group to form 6:2 FTSA. 6:2 FTSA was further desulfonated and oxidized to produce alcohol, aldehyde and then 6:2 FTUCA and 6:2 FTCA, which underwent removing -HF, and CH_2_-CH_2_, CH_2_-CF_2_, and CF_2_-CF_2_ bonds break through the β-oxidation pathway. Finally, the stable terminal product of PFCA was formed. PFHpA was not detected in this process, indicating that α-oxidation did not occur. Consistent with our previous findings on 6:2 FTSA biotransformation in earthworms, we demonstrated that this downstream product of 6:2 FTAB underwent similar bioaccumulation and β-oxidation pathways, yielding analogous metabolites (PFHxA, PFPeA, PFBA, PFPrA, and TFA) [15]. But it was worth noting that TFA was only detected in the 6:2 FTSA-based metabolization study, and the degradation of 6:2 FTAB in earthworms was much slower than 6:2 FTSA. These compound-specific distinctions suggested the betaine moiety in 6:2 FTAB sterically might hinder complete oxidation, arresting degradation at earlier stages.

### 3.4. Biodegradation of 6:2 FTAB by Earthworm Gut Microorganisms

The gut of earthworms exposed to organic pollutants in soil contains a large population of aerobic and anaerobic bacterial communities capable of degrading pollutants. To further study the contribution of gut bacteria to metabolism of 6:2 FTAB in earthworms, gut microbes were isolated from earthworm gut for aerobic and anaerobic degradation experiments by simulating the earthworm gut environment in the laboratory.

**Figure 4:**
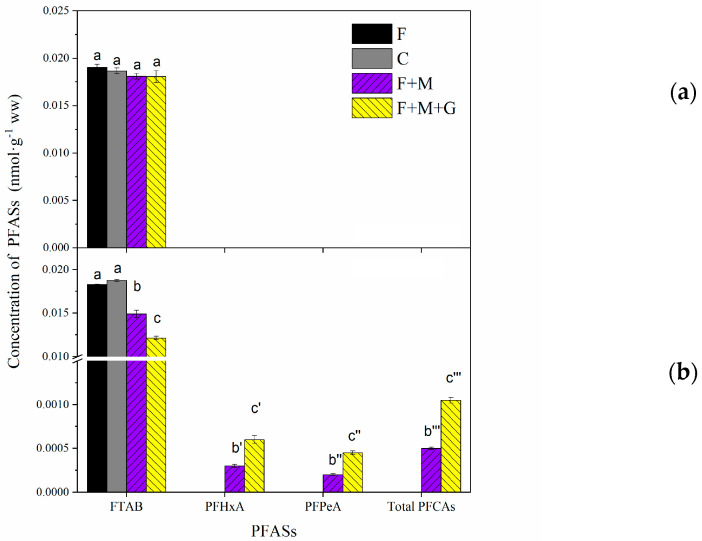
(**a**) Effects of gut anaerobic bacteria and (**b**) aerobic bacteria on biodegradation of 6:2 FTAB. F: concentration of 6:2 FTAB in blank control group; C: test group for observing the abiotic interference with boiled gut microbiota; F + M: gut microbiota cultured with 6:2 FTAB medium; F + M + G: gut microbiota spiked culture with 6:2 FTAB and addition of 0.5% glucose. Different letters indicate significant differences (*p* < 0.05).

Figure 4a showed the degradation of 6:2 FTAB by anaerobic microbes in the earthworm gut. The concentrations of 6:2 FTAB displayed no significant changes in all treatment groups (F, F + M, F + M + G) and controls (C), suggesting that the contribution of gut-associated anaerobic bacteria to 6:2 FTAB biodegradation in earthworms was negligible. However, as shown in Figure 4b, the concentrations of 6:2 FTAB in the F + M and the F + M + G groups are significantly decreased (*p* < 0.05), while terminal PFCA degradation products (PFHxA and PFPeA) are significantly increased, suggesting the strong contribution of gut aerobic bacteria to the 6:2 FTAB biodegradation in earthworms. Otherwise, compared to the initial concentration of 6:2 FTAB (0.019 nmol g^−1^) in the aerobic environment of the F group, no significant change was observed in the abiotic C group, indicating that abiotic interference was negligible during the experiment (Figure 4b). The molar distribution profiles of PFCAs in F + M and F + M + G groups were both as follows: PFHxA > PFPeA. Many previous studies have reported that gut microbes could promote the degradation of pollutants, such as tetrabromodiphenyl ether (BDE47) [26], hexachlorocyclohexan and endosulfan [27,28]. However, our previous study found that the mixed bacteria isolated from the earthworm gut exhibited insignificant direct effects on the biodegradation of PFOSA [16]. Additionally, the concentrations of PFCAs’ degradation products (PFHxA, PFPeA and total PFCAs) in the F + M + G group were significantly higher than in the F + M group, indicating that glucose acting as a co-metabolic substrate influenced the symbiotic network of aerobic microbes and enhanced the degradation of 6:2 FTAB. Overall, the presence of gut-associated aerobic bacteria contributed to the biodegradation of 6:2 FTAB in earthworms. The observation of PFHxA and PFPeA implied that the *β*-oxidation of 6:2 FTAB by gut-associated microorganisms did occur, rather than α-oxidation, which was similar to that for earthworms.

### 3.5. Oxidative Stress of Earthworms in Response to 6:2 FTAB

**Figure 5:**
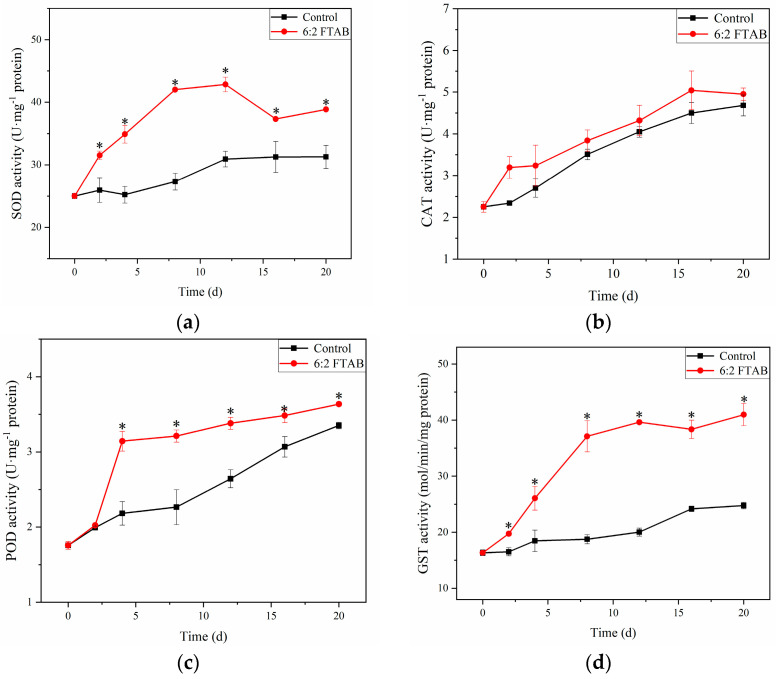
(**a**)The effects of 6:2 FTAB on activities of (**a**) SOD, (**b**) CAT, (**c**) POD and (**d**) GST in earthworms (* *p* < 0.05).

To prevent oxidative damage caused by external environmental pollutants, organisms have developed a series of antioxidant defense systems, including enzymatic and nonenzymatic systems, which are crucial for eliminating oxygen free radicals produced under oxidative stress [29]. The main enzymatic defense systems in earthworms consist of SOD, CAT and POD, while glutathione (GSH) belongs to non-enzymatic antioxidant defense systems that detoxify oxygen free radicals [30]. The SOD, POD and CAT enzymes prevent the reactive oxygen species (ROS) generation, which are involved in the detoxification of free radical (O^2−^) and hydrogen peroxide (H_2_O_2_), respectively [21]. It is well known that GST exerts its antioxidative activity by catalyzing the attachment of GSH to a range of electrophilic species for the elimination of toxic xenobiotics to protect cells against oxygen free radicals and detoxification of xenobiotics [31]. The antioxidant responses of earthworms induced by 6:2 FTAB were investigated by analyzing biomarkers of oxidative stress in earthworms (Figure 5a–d). Except for CAT, the activities of SOD, POD and GST in earthworms exposed to 6:2 FTAB over the duration of the exposure were significantly increased by 21.5~53.8%, 8.4~43.9%, 41.4~97.0% activation compared with the controls, respectively (*p* < 0.05), suggesting that these enzymes in earthworms were sensitive to 6:2 FTAB. The enhancement of these antioxidant enzyme activities also indicated that the oxidative stress in earthworms resulted from 6:2 FTAB, while the antioxidant defense system was activated to scavenge the excessive ROS induced by 6:2 FTAB.

Previous studies have reported the oxidative stress in earthworms and other animals induced by PFAS compounds. Our previous study found that accumulated of N-ethyl perfluorooctane sulfonamide ethanol (N-EtFOSE) significantly increased the activities of CAT, SOD and POD, which demonstrated that N-EtFOSE had the potential to induce oxidative stress in earthworms [30]. It was found that the activities of SOD, POD and CAT in earthworms were initially activated and then inhibited under PFOS stress, suggesting that PFOS induced oxidative stress [32]. It was reported that the activity of SOD was significantly promoted, while the activity of CAT was altered insignificantly in the liver of animals exposed to N-EtFOSE [33]. But our previous study found the obvious inhibition of SOD, CAT, POD and GST activities in plants, suggesting the damage of antioxidant defense systems and a failure to mediate detoxification of 6:2 FTAB in plants [11]. Zhang et al. [34] reported that GST was involved in biotransformation of 8:2 fluorotelomer alcohol (8:2 FTOH) in plants through the formation of glutathione conjugates [34].

SOD can catalyze the dismutation of superoxide radical (O^2−^) to H_2_O_2_ and O_2_. POD and CAT are known to be responsible for the detoxification of H_2_O_2_ produced by SOD to earthworm cells [24]. Thus, it was inferred that the free radical (O^2−^) in the earthworms stimulated the SOD activity, and the increase of POD could eliminate H_2_O_2_ produced by SOD in earthworm cells. Thus, the activation of SOD, POD and GST activities suggested the antioxidant system and GST played positive roles against 6:2 FTAB oxidative stress and phase II detoxification of 6:2 FTAB in earthworms, respectively.

## 4. Conclusions

Our study demonstrated that 6:2 FTAB could be degraded in soil to form PFHxA, PFPeA, PFBA and PFPrA. Earthworms exhibited the efficient bioaccumulation of 6:2 FTAB from soil, with subsequent in vivo and in vitro biotransformation yielding PFHxA, PFPeA, PFBA and PFPrA. We observed that the aerobic bacteria isolated from earthworm gut could biodegrade 6:2 FTAB to form PFHxA and PFPeA, while anaerobic bacteria was not prone to degrade 6:2 FTAB. All these results suggested that the metabolization of 6:2 FTAB did occur in earthworm homogenate and gut bacteria through *β*-oxidation. The activities of SOD, POD and GST were significantly increased by 6:2 FTAB, while CAT activities were not changed, indicating that the antioxidant system and GST played positive roles against 6:2 FTAB oxidative stress and phase II detoxification of 6:2 FTAB in earthworms, respectively. This study contributed to the understanding of the environmental risk of 6:2 FTAB in terrestrial invertebrates.

## Data Availability

Data are available in the article/Supplementary Materials and on request from the corresponding author.

## Supplementary Materials

The following supporting information can be downloaded at: https://www.mdpi.com/article/10.3390/toxics13050337/s1, Chemical extraction and analysis of soil, earthworm and gut bacteria incubation samples; enzyme assays; instrumental analysis; quality assurance and quality control; Table S1. List of PFASs monitored in the present study, the optimized UPLC-MS/MS parameters, the recoveries (%) and the method detection limits (MDLs, 3:1 S/N) in earthworm homogenate (pmol g^−1^), earthworm gut microbe incubation (pmol mL^−1^) and soil (pmol g^−1^); Figure S1. Concentrations of 6:2 FTAB and PFCAs in control groups (nmol g^−1^ ww). Values are mean ± SD, *n* = 3. Control I (homogenate without 6:2 FTAB); control II (6:2 FTAB + homogenates inactivated by boiling at 100 °C for 5 min, excluding the interference of abiotic or microbial); control III (6:2 FTAB, excluding the photodegradation). Samples collected after 2 h and 32 h of incubation. References [35,36,37] are cited in Supplementary Materials.
