# Supplementary material for: Bioaccumulation, Biotransformation and Oxidative Stress of 6:2 Fluorotelomer Sulfonamidoalkyl Betaine (6:2 FTAB) in Earthworms (Eisenia fetida)
Source: Toxics. 2025 Apr 24;13(5):337. doi: 10.3390/toxics13050337 (PMC12115925; doi:10.3390/toxics13050337)
Supplement: Supplementary file 1 [file toxics-13-00337-s001.zip › toxics-3573000-supplementary.doc]

Supporting Information

*for*

**Bioaccumulation, biotransformation and oxidative stress of 6:2 fluorotelomer sulfonamidoalkyl betaine (6:2 FTAB) in earthworms (*Eisenia fetida*)**

Xinlei Zhang , Mengyao Fang , Zhiyuan Bai , Yulu Zong, Shuyan Zhao^[1]*^, Jingjing Zhan

Key Laboratory of Industrial Ecology and Environmental Engineering，Ministry of Education, School of chemical engineering, Ocean and Life science, Dalian University of Technology, Panjin, Liaoning, P.R. China 124221

**1. Chemical extraction and analysis of soil, earthworm and gut bacteria** **incubation samples**

**Soil.** The extraction method for soil samples was similar to our previous methods for soil (Zhao et al., 2017). The collected soil samples were freeze-dried for 24 hours. Approximately 1 g of dried soil was weighed into a 10 mL polypropylene (PP) centrifuge tube, and 5 mL of methanol was added, followed by shaking at 250 rpm for 10 minutes. The tubes were then placed in an ultrasonic bath at 40 °C for 10 minutes. The mixture was centrifuged at 3000 rpm for 10 minutes. This extraction process was repeated three times, and the supernatants were combined into a new PP tube. The combined supernatant was evaporated to approximately 5.0 mL under a gentle stream of nitrogen. The cleanup process using Clearnert Pesticarb-SPE cartridges (250 mg/6 mL, Agela Technologies) followed the method described in our previous study (Zhao et al., 2014).

**Earthworm and gut bacteria incubation samples.** The extraction of PFASs from earthworms and the further extraction of gut bacteria culture followed the steps outlined in a previous study (Zhao et al., 2018). Briefly, 0.1 g (wet weight) of homogenate or 1 mL of gut bacteria culture solution was weighed in a 15-mL polypropylene (PP) tube, followed by the addition of 1 mL of 0.5 M TBAHS (adjusted to pH 10 with sodium hydroxide) and 2 mL of 0.25 M sodium carbonate buffer. The mixture was vortexed thoroughly, and then 5 mL of MTBE was added for extraction. The mixture was vortexed and shaken at 250 rpm for 20 minutes at room temperature, followed by centrifugation at 6000 rpm for 10 minutes. The organic MTBE layer was separated from the aqueous layer and collected into a new PP tube. The extraction procedure was repeated three times, and the MTBE fractions were combined into the same tube. The combined MTBE was then evaporated to near dryness under a gentle stream of nitrogen. Subsequently, 3.0 mL of methanol was added to the residue and vortexed to reconstitute the sample. The extract was purified using a Clearnert Pesticarb-SPE cartridge (500 mg/6 mL, Agela Technologies) following the method described in a previous study (Zhao et al., 2014). The SPE cartridge was conditioned with 5 mL of 0.1% NH4OH in methanol, 5 mL of water, and 5 mL of methanol. Then, 3 mL of the extract was passed through the preconditioned cartridge at a rate of 1 drop per second, and the eluate was collected in a PP tube. The cartridge was further eluted with 5 mL of methanol, which was also combined into the same tube. The collected solution was evaporated under a gentle stream of nitrogen to approximately 1 mL of methanol. The extracts were centrifuged at 12000 g for 20 minutes, and 1 mL of the supernatant was transferred into an autosampler vial and stored at -20°C prior to UPLC/MS/MS analysis.

**2.** **Enzyme assays**

The activities of SOD, POD, CAT, and GST were measured using earthworm ELISA kits (Dongge Biotechnology Co. Ltd, Beijing, China) on a Multi-Mode Microplate Reader (Molecular Device, USA). Following the instructions, the enzyme extraction solution was thawed before analysis. The assay procedure was as follows: the samples were diluted, and HRP-conjugate reagent was added to each well except the blank well. The plate was covered with an adhesive strip and incubated at 37°C for 60 minutes. The liquid was then removed. Chromogen solutions A and B were added to each well and incubated at 37°C for 15 minutes. The optical density (O.D.) was read using a microplate reader within 15 minutes after adding the stop solution.

**3. Instrumental Analysis**

The PFASs in the samples were separated and quantified using a Waters Ultra Performance Liquid Chromatography system coupled with a Waters XEVO-TQS tandem mass spectrometry (UPLC-MS/MS), operating in negative electrospray ionization (ESI) mode. The injection volume was 10 μL, and the mobile phases consisted of 2 mmol/L ammonium acetate (A) and HPLC-grade methanol (B). The LC separation conditions were as follows: starting at 25% B for 0.5 minutes, followed by a gradient increase to 85% B at 5.0 minutes, then to 100% B at 5.1 minutes, and held until 7 minutes before returning to the initial conditions at 10 minutes. The flow rate was 0.3 mL/min. Separation of PFASs was achieved using a Waters UPLC C18 column (1.7 μm, 2.1 mm × 50 mm) at 38°C. Electrospray ionization was operated in negative mode (ESI), and multiple reaction monitoring (MRM) mode was used for all data analysis. The MS parameters were as follows: capillary voltage, 2.0 kV; nebulizer gas flow, 7.00 bar; desolvation gas flow, 800 L/h; cone gas flow, 150 L/h; and desolvation temperature, 400°C. The mass transitions, cone voltages, and collision energies for PFASs are listed in Table S1.

**4. Quality assurance and quality control**

PFAS standards were added to the blank matrix to assess quality assurance. Quality control was performed to monitor potential method contamination by regularly analyzing procedural blanks and injecting solvent blanks and standards alongside every 12 real samples. The blank matrix was obtained from earthworms cultured in clean soil without the addition of PFASs. The method detection limit (MDL) was determined based on matrix samples with a signal-to-noise ratio of 3:1 (3:1 S/N) if specific PFASs were not detected in the earthworm and soil blanks. Recoveries were determined by spiking known amounts of PFAS standards into clean whole earthworm homogenate (10, 40, and 80 ng/g wet weight) and soil (10, 40, and 80 ng/g dry weight).

**5. Biodegradation of 6:2 FTAB in soil**

Biodegradation kinetic constant (*k*_s_) and degradation half-life (*t_1/2_*) of 6:2 FTAB in soil were calculated as:

 (S1)

= (S2)

where *C_S_* is 6:2 FTAB concentration (nmol/g dw) in soil at time *t* (d), *C_S0_* is the initial concentration of 6:2 FTAB spiked in soil (nmol/g dw), and *k_s_* is the degradation rate constant of 6:2 FTAB in soil (1/d).

**6. Biodegradation of 6:2 FTAB in earthworm homogenate**

The biodegradation rate constant (*k_h_*) of 6:2 FTAB in earthworm homogenates was calculated as:

 (S3)

Where *C_h_* is 6:2 FTAB concentration in earthworm homogenates (nmol/g ww) at time *t* (h), and *C_0_* is 6:2 FTAB concentration at beginning of the incubation.

**References**

Zhao, S., Fang, S., Zhu, L., Liu, L., Liu, Z., Zhang, Y., 2014. Mutual impacts of wheat (*Triticum aestivum* L.) and earthworms (*Eisenia fetida*) on the bioavailability of perfluoroalkyl substances (PFASs) in soil. Environmental Pollution 184, 495-501.

Zhao, S., Wang, B., Zhu, L., Liang, T., Meng, C., Yang, L., Lv, J., Liu, L., 2018. Uptake, elimination and biotransformation of N-ethyl perfluorooctane sulfonamide (N-EtFOSA) by the earthworms (*Eisenia fetida*) after *in vivo* and *in vitro* exposure. Environmental Pollution 241, 19.

Zhao, S.; Zhu, L., 2017. Uptake and metabolism of 10:2 fluorotelomer alcohol in soil-earthworm (*Eisenia fetida*) and soil-wheat (*Triticum aestivum* L.) systems. Environmental Pollution 220, 124-131.

**Table S1** List of PFASs monitored in the present study, the optimized UPLC-MS/MS parameters, the recoveries (%) and the method detection limits (MDLs, 3:1 S/N) in earthworm homogenate (pmol/g ww), earthworm gut microbe incubation (pmol/mL) and soil (pmol/g).

Table S1. Recoveries (%) and method detection limit (MDL) of PFASs in soil (pmol/g dw), earthworm (pmol/g ww) and gut microbe incubation (pmol/mL)

| **Compound** | **Monitoring transition** | **Monitoring transition** | **Cone voltage (V)** | **Earthworm homogenate** | | **Soil** | | **Gut microbe incubation** | |
| --- | --- | --- | --- | --- | --- | --- | --- | --- | --- |
|  |  |  |  | **Recovery** | **MDL** | **Recovery** | **MDL** | **Recovery** | **MDL** |
| 6:2 FTAB | 376.93→292.88 | 50.0 | 28.0 | 107±6.72 | 0.0009 | 81.5±6.72 | 0.0035 | 102±7.30 | 0.0012 |
| PFPrA | 163.00→119.00 | 15.0 | 10.0 | 103±4.83 | 0.0020 | 85.7±3.12 | 0.0075 | 97.5±4.02 | 0.0021 |
| PFBA | 213.00→169.00 | 18.0 | 8.0 | 103±1.52 | 0.0010 | 94.2±1.06 | 0.0022 | 101±2.60 | 0.0013 |
| PFPeA | 263.00→219.00 | 19.0 | 8.0 | 95.3±8.28 | 0.0003 | 82.4±3.33 | 0.0028 | 97.8±2.13 | 0.0029 |
| PFHxA | 313.00→269.00 | 12.0 | 9.0 | 99.7±0.79 | 0.0001 | 87.1±6.35 | 0.0005 | 103±1.51 | 0.0015 |

**Figure S1.** Concentrations of 6:2 FTAB and PFCAs in control groups (nmol/g ww). Values are mean ± SD, n = 3. Control I (homogenate without 6:2 FTAB); control II (6:2 FTAB + homogenates inactivated by boiling at 100°C for 5 min, excluding the interference of abiotic or microbial); control III (6:2 FTAB, excluding the photodegradation). Samples collected after 2 h and 32 h of incubation.

1. ^^ Corresponding author, E-mail address: [zhaoshuyan@dlut.edu.cn](mailto:zhaoshuyan@dlut.edu.cn). Phone: +86-427-2631790
